# Supplementary material for: OspA antibodies inhibit the in vitro transmigration of Borrelia burgdorferi
Source: Infect Immun. 2026 Apr 21;94(5):e00128-26. doi: 10.1128/iai.00128-26 (PMC13163200; doi:10.1128/iai.00128-26)
Supplement: Supplemental figures — Fig. S1 to S5. [file iai.00128-26-s0001.pdf]

Supplementary Methods

Dark-field Microscopy

For examination of the spirochetes in the lower chamber of the Transwell units, 7 µL aliquots were spotted onto glass microscope slides and covered with cover glass (22×22, No. 1, 0.13-0.16 mm thickness). Spirochetes were imaged using an Olympus BH-2 RFCA upright microscope (Evident Scientific; Waltham, MA) paired with a 20X objective (N.A. 0.70) in a dark-field setting. Images and videos were acquired using an Excelis™ MPX-20RC microscopy camera (Accu-Scope, Commack, NY) paired with CaptaVision+ software version 2.4.9.0 for image analysis.

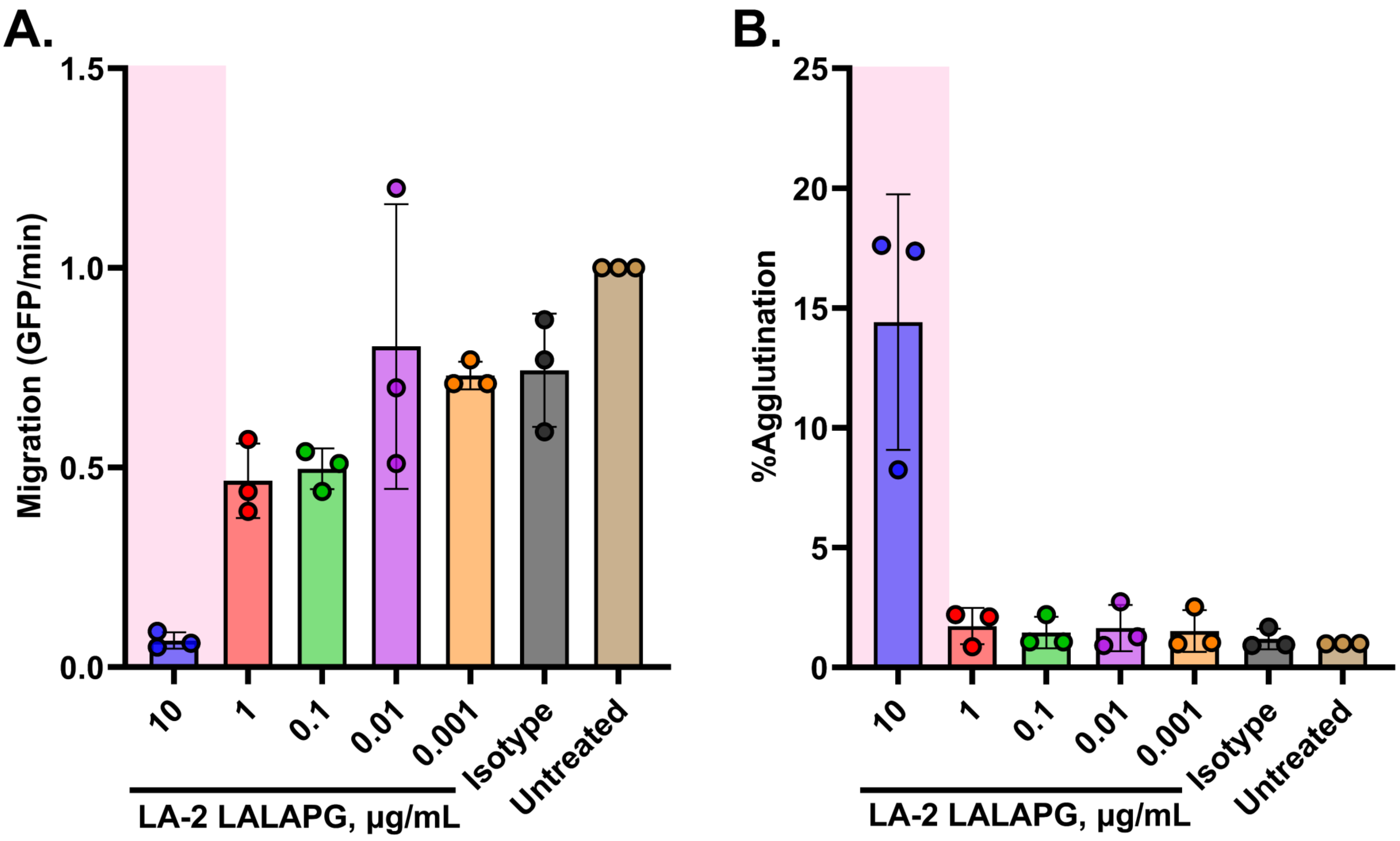

Figure S1. A Fc-silent variant of LA-2 (LA-2 LALAPG) inhibits the migration of *B. burgdorferi* B31-A in a dose-dependent manner. $2{\times}10^7$ GFP-tagged spirochetes were either untreated or treated with a range of LA-2 LALAPG concentrations or 10 µg/mL of isotype control mAb in the Transwell lower chamber. The Transwell migration assay was performed as described in Materials and Methods. Data was acquired from three independent biological replicates. **(A)** Data represents migration (GFP events/min) normalized to untreated spirochetes and error bars represent standard deviation of the mean. (B) Data represents percent agglutination of spirochetes in the lower Transwell chamber normalized to untreated spirochetes and error bars represent standard deviation of the mean. Statistical significance was determined by one-way ANOVA followed by Dunnett's *post hoc* multiple comparisons test. Pink shading indicates $p \leq 0.05$ and compared to isotype mAb-treated group.

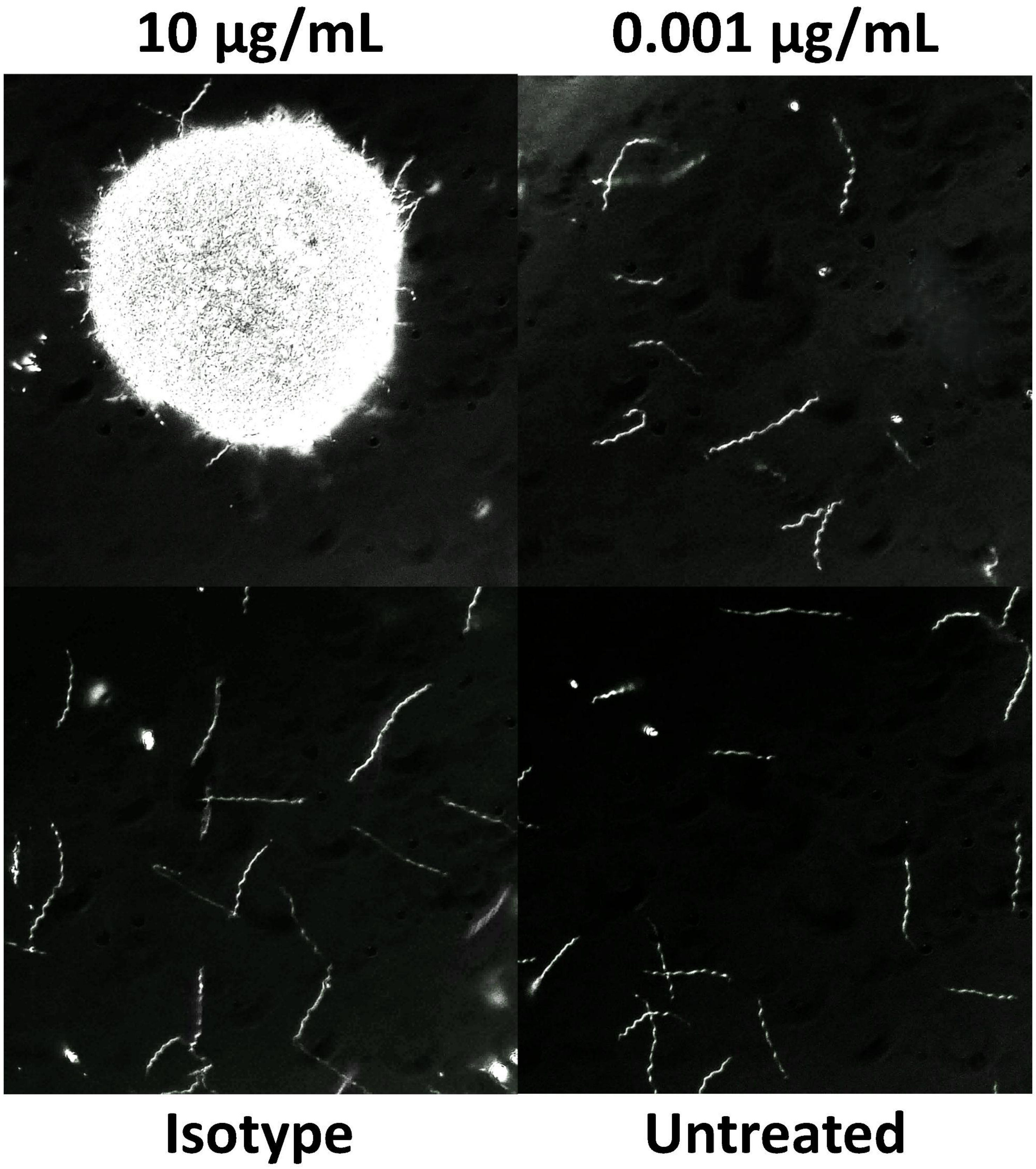

Figure S2. Dark field microscopy images of *B. burgdorferi* B31-A exposed to varying concentrations of LA-2. Representative images show spirochetes treated with the highest and lowest concentration of LA-2 in the dose response range, or in control conditions in the Transwell assay. All images have been captured at 20X objective magnification (Refer to Supplementary Methods).

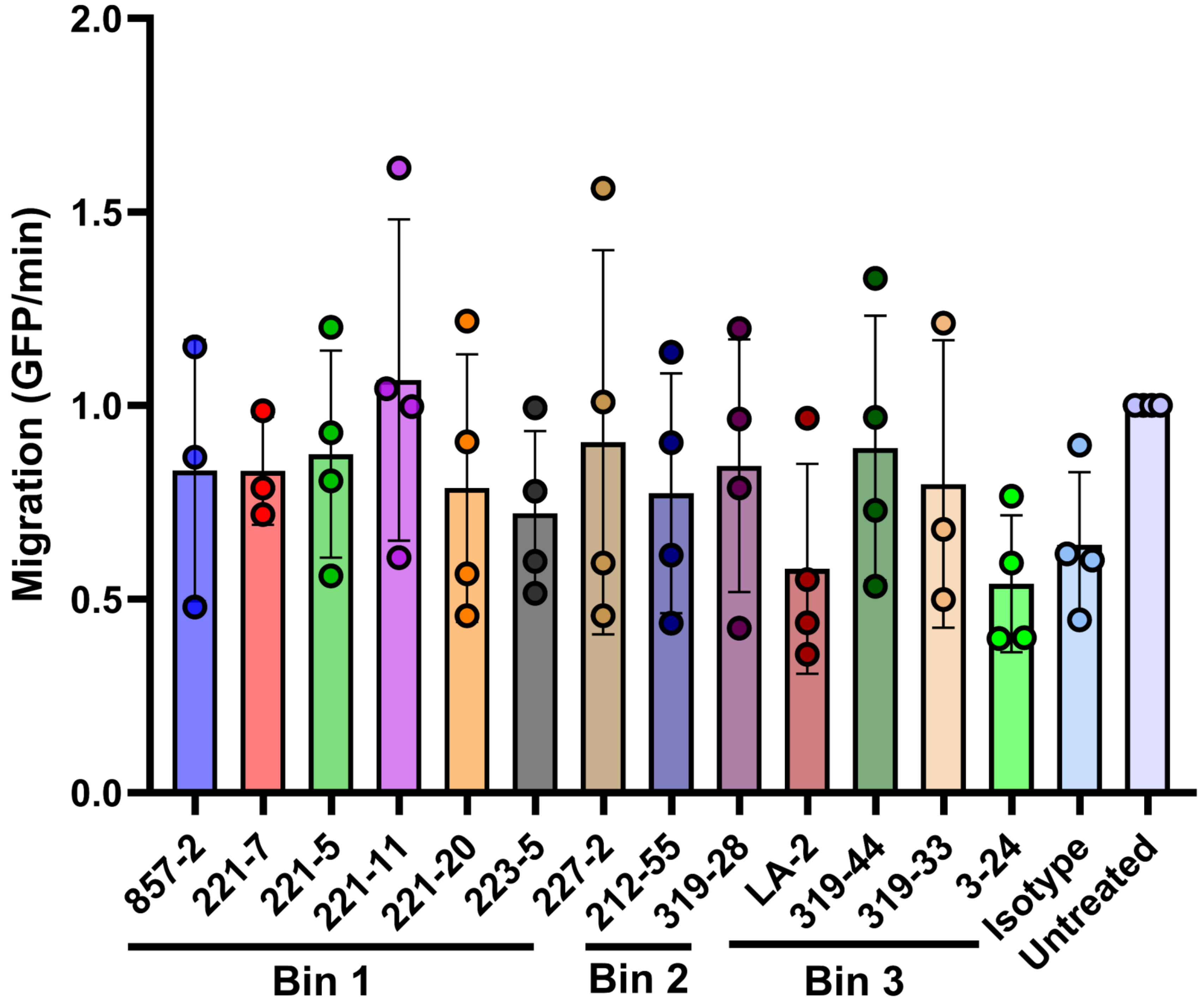

Figure S3. A panel of OspA mAbs fail to inhibit the migration of *B. burgdorferi* B31-A at a low dose. 2×10^{7} GFP-tagged spirochetes were either untreated or treated with 1 µg/mL of individual OspA mAbs or an isotype control mAb in the Transwell assay (Refer to Materials and Methods). Data was acquired from three independent biological replicates. Data represents migration (GFP events/min) normalized to untreated spirochetes and error bars represent standard deviation of the mean. There was no statistically significant difference compared to isotype mAb-treated group, as determined by one-way ANOVA followed by Dunnett's *post hoc* multiple comparisons test.

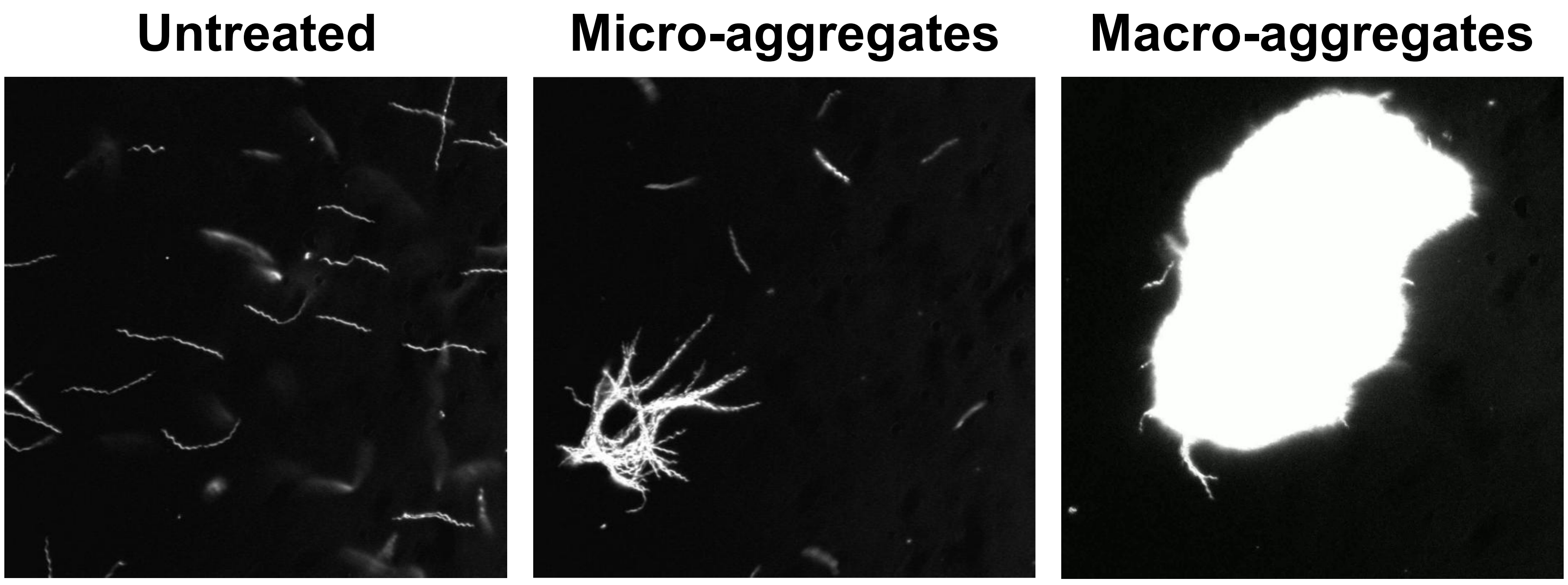

Figure S4. Dark field microscopy images of *B. burgdorferi* B31-A exposed to different OspA mAbs. Representative images show untreated spirochetes and the formation of micro-aggregates, mostly by Bin 1 and Bin 2 mAbs, and macro-aggregates, mostly by Bin 3 mAbs, with a few exceptions. All images have been captured at 20X objective magnification (Refer to Supplementary Methods).

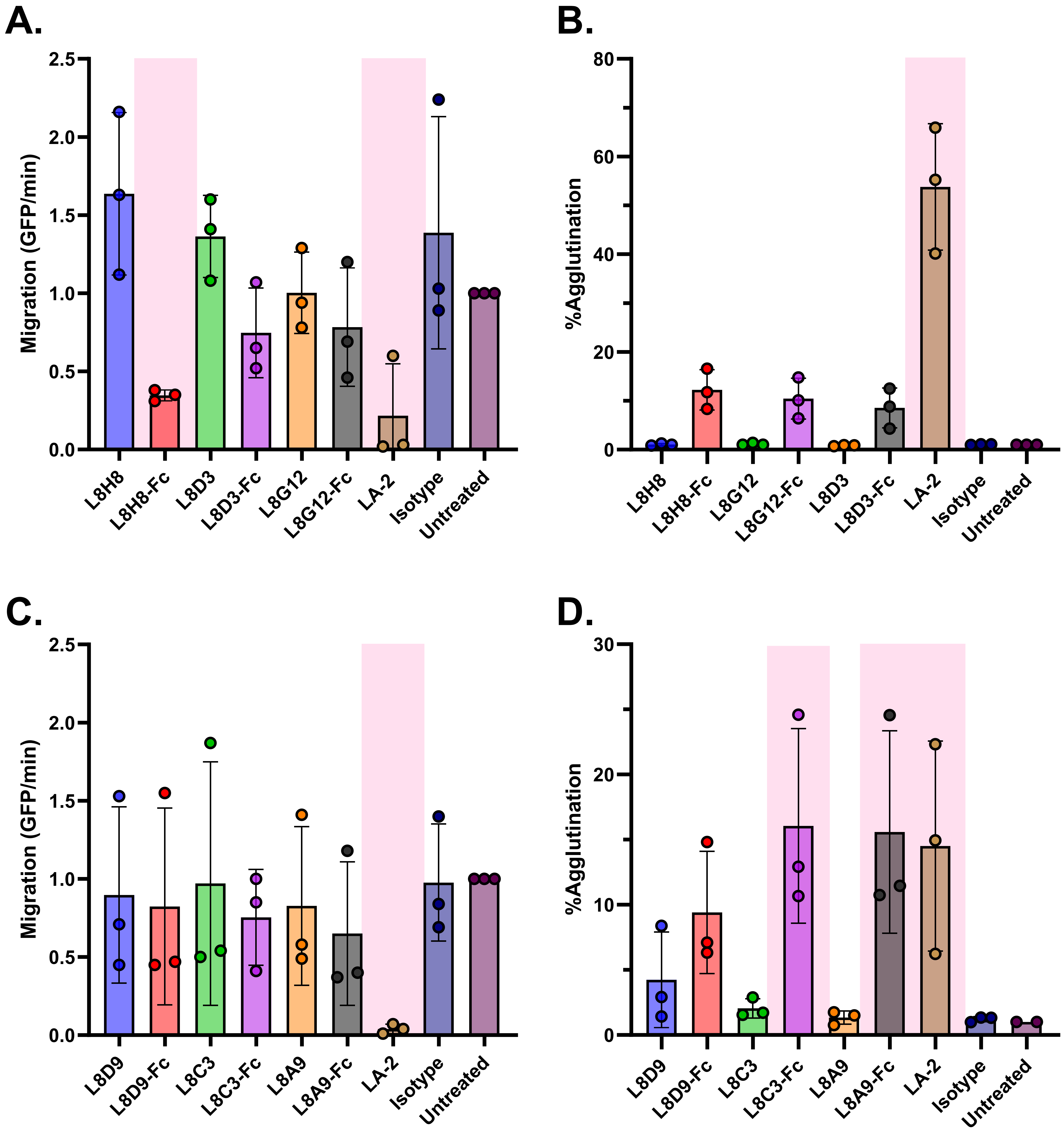

Figure S5. Impact of Bin 1 and Bin 3 representative V_HH and V_HH-IgG constructs on the migration of *B. burgdorferi* B31-A and its correlation with spirochete agglutination. $2×10^7$ GFP-tagged spirochetes were either untreated or treated with 10 μg/mL of either V_HH or V_HH-IgGs or an isotype control mAb in the Transwell lower chamber. LA-2 at 10 μg/mL was used as the positive control. The Transwell migration assay was performed as described in Materials and Methods. Data were acquired from three independent biological replicates. **(A, C)** Data represent migration (GFP events/min) of spirochetes treated with Bin 1 and Bin 3 V_HH and V_HH-IgGs, respectively, normalized to untreated spirochetes, and error bars represent standard deviation of the mean. **(B, D)** Data represent percent agglutination of spirochetes treated with Bin 1 and Bin 3 V_HH and V_HH-IgGs, respectively, in the lower Transwell chamber normalized to untreated spirochetes and error bars represent standard deviation of the mean. Statistical significance was determined by one-way ANOVA followed by Dunnett's *post hoc* multiple comparisons test. Pink shading indicates $p \leq 0.05$ when compared to isotype mAb-treated group.
